# Evidence for carrier localization in the pseudogap state of cuprate superconductors from coherent quench experiments

**DOI:** 10.1038/ncomms7958

**Published:** 2015-04-20

**Authors:** I. Madan, T. Kurosawa, Y. Toda, M. Oda, T. Mertelj, D. Mihailovic

**Affiliations:** 1 Jozef Stefan Institute and International Postgraduate School, Jamova 39, SI-1000 Ljubljana, Slovenia; 2 Department of Physics, Hokkaido University, Sapporo 060-0810, Japan; 3 Department of Applied Physics, Hokkaido University, Sapporo 060-8628, Japan

## Abstract

A ‘pseudogap' was introduced by Mott to describe a state of matter that has a minimum in the density of states at the Fermi level, deep enough for states to become localized. It can arise either from Coulomb repulsion between electrons, and/or incipient charge or spin order. Here we employ ultrafast spectroscopy to study dynamical properties of the normal to pseudogap state transition in the prototype high-temperature superconductor Bi_2_Sr_2_CaCu_2_O_8+*δ*_. We perform a systematic temperature and doping dependence study of the pseudogap photodestruction and recovery in coherent quench experiments, revealing marked absence of critical behaviour of the elementary excitations, which implies an absence of collective electronic ordering beyond a few coherence lengths on short timescales. The data imply ultrafast carrier localization into a textured polaronic state arising from a competing Coulomb interaction and lattice strain, enhanced by a Fermi surface instability.

## Abstract (web summary)

Understanding the nature of the pseudogap phase is an important challenge for high-temperature superconductivity. Madan *et al*. present a series of ultrafast spectroscopy experiments on the pseudogap phase of Bi_2_Sr_2_CaCu_2_O_8+δ_ and find evidence of carrier localization into a textured polaronic state.

## Graphical abstract

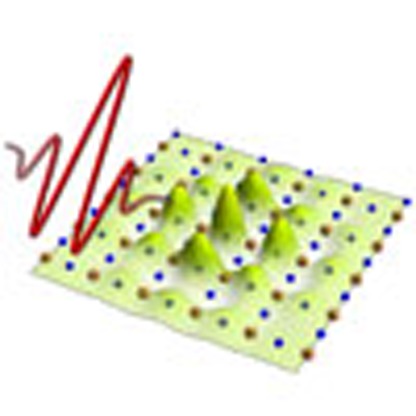

The PG appears in many different systems of current interest^1^,^2^,^3^, attracting attention partly due to its resulting unusual physical properties, and partly because it is commonly associated with the emergence of a long-range ordered (LRO) broken symmetry state, such as superconductivity (SC) or a charge or spin density wave order^4^,^5^,^6^,^7^,^8^. Understanding its origin in the particular case of high-temperature superconductors is often thought to be of primary importance for determining the mechanism of high-temperature superconductivity.

In the absence of thermodynamic evidence of any phase transitions associated with the formation of the PG state^9^, its origin was considered to be due to a fluctuating or short-range crossover phenomenon originating either from localized carriers in the form of polarons (or clusters of polarons organized in stripes)^10^,^11^ or fluctuating spin or charge-density waves (SDW or CDW)^12^,^13^,^14^. In the latter case the gap in the low-energy spectrum is formed by a periodic potential, while in the former case the energy scale associated with the PG is related to the binding energy of the localized states. There are, however, clear spectroscopic evidences for the reduction of fourfold to twofold symmetry^15^,^16^ and time-reversal^17^ symmetry breaking associated with the onset of the pseudogap. These experiments, though being indicative of a phase transition, did not reveal any characteristic length scale or the origin of the PG excitations; hence, it was not clear whether the broken symmetry state is long-range ordered or symmetry is broken only locally by objects such as polarons.

Recently, advanced resonant X-ray studies^12^,^18^ have indicated the presence of short-range charge modulation in the bulk material, complementary to numerous tunnelling microscopy studies of surface charge order associated with the pseudogap formation in the Bi_2_Sr_2_CaCu_2_O_8+*δ*_ (Bi2212) family. The feature seems to be universal and is quite different in nature from static stripes observed in La_2−*x*_Ba_*x*_CuO_4_ (LBCO)^19^, having a different doping dependence of the characteristic wavevector. Importantly the onset of charge correlations agrees with the onset of the pseudogap phase through the phase diagram, unlike that reported for static stripes around 1/8 doping.

Ultrafast optical techniques were shown to be able to detect fluctuating superconducting^20^ or charge density wave^14^ orders particularly in La_1.9_Sr_0.1_CuO_4_ (ref. 14; where CDW possesed long enough correlations to show collective modes). For CDW systems this can be explained by the sensitivity of ultrafast optical techniques to the electronic degrees of freedom that on short timescales can behave quite distinctly from the lattice^21^. This might be the case also for the pseudogap state. Here we utilize ultrafast coherent quench spectroscopy as a recently developed and appropriate tool for a systematic study of the quasiparticle (QP) dynamics associated with the photodestruction of the pseudogap state and its consequent recovery in a non-equilibrium transition.

For gapped laser-excited systems evolving through a transition in time, the QP lifetime *τ*_QP_ exhibits critical behaviour, diverging as *τ*_QP_∼1/Δ as *t*→*t*_c_ where *t*_c_ is defined as the time when the order parameter becomes non-zero and the gap Δ=Δ(*t*−*t*_c_) opens. Examples of dynamical phase transition measured in three-pulse coherent quench experiments include both quasi-1D and quasi-2D CDW systems: TbTe_3_, DyTe_3_, 2H-TaSe_2_, K_0.3_MoO_3_ (ref. 22) and superconducting La_2−*x*_Sr_*x*_CuO_4_ near 1/8 doping^23^. The QP lifetime divergences associated with the superconducting transition under quasi-equilibrium conditions are observed ubiquitously in cuprates (YBa_2_Cu_3_O_7−*x*_ (ref. 24), La_2−*x*_Sr_*x*_CuO_4_ (ref. 25), Bi_2_Sr_2_CaCu_2_O_8+*δ*_ (ref. 26), HgBa_2_Ca_2_Cu_3_O_8_ (ref. 27), YBa_2_Cu_4_O_*y*_ (ref. 28)) and pnictides^29^. A similar divergence is also observed at the SDW transition in pnictides^30^,^31^.

In the pseudogap case, pump-probe experiments show a broad transition^25^,^26^,^28^ exhibiting the well known problem of the definition of T* common to different techniques. These quasiequilibrium experiments show no evidence for any divergence at T* either due to smearing or intrinsic short-range character of the excitations. However, in coherent quench experiments smearing of the transition due to inhomogeneity in *T*_c_*s* is absent, since the gap opens simultaneously throughout the entire excited volume. Such experiments may thus reveal the presence of a phase transition even in the case of spatial inhomogeneity. If the pseudogap is associated with long-range ordered symmetry breaking transition this should show up as a divergence of the quasi-particle response.

In the following we present a study of the complete suppression and recovery of the pseudogap state in Bi_2_Sr_2_CaCu_2_O_8+*δ*_ for doping levels varying from underdoped (UD) to overdoped (OD) (See Methods) at different temperatures above the superconducting *T*_c_ and discuss the results in the context of possible origin of the pseudogap state (Fig. 1). We show that the pseudogap is destroyed in the result of initial hot electron scattering and its photodestruction energy is defined mostly by the energy lost to the subgap phonons. The clear abscence of the divergence of the quasiparticle relaxation at the reappearance of the pseudogap response is indicative of the short-range nature of the state, whereas exponential character of the recovery with weak dependence of the characteristic time on fluence and temperature suggests localization of independent carriers as relaxation mechanism.

## Results

### Photodestruction of the pseudogap state

**Figure 2:**
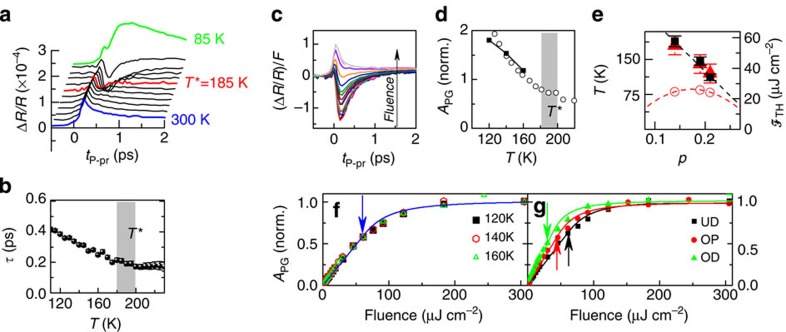
Pseudogap response as a function of temperature and fluence. (**a**) A waterfall plot of Δ*R*/*R* as a function of pump-probe delay in a temperature region from 85 to 300 K. (**b**) Relaxation time of the negative pseudogap component as a function of temperature. (**c**) Transient reflectivity normalized by the incident fluence 
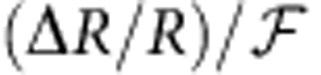
 for the underdoped sample at 120 K. At low excitation the response is linear and the curves overlap. At low excitation the negative pseudogap component is dominant, whereas at high excitation it is saturated and hot-electron energy relaxation component is prevailing. (**d**) Amplitude of the saturated pseudogap signal (Δ*R*_SAT_/*R*) as a function of temperature (black squares). For comparison renormalized low fluence temperature dependence of the amplitude from ref. 26 is plotted (open circles). (**e**) The value of photodestruction threshold fluence (black squares) as a function of doping. For comparison doping dependence of T* (red triangles) and *T*_c_ (red circles) are shown in the same graph. (**f**) Normalized fluence dependence of the amplitude of the pseudogap component at different temperatures for the underdoped sample, the threshold fluence is marked by an arrow. (**g**) Normalized fluence dependence of the amplitude of the pseudogap component for different doping levels: underdoped (UD) at 120 K, optimally doped (OP) at 120 K and overdoped (OD) at 110 K, the threshold fluence is marked by arrows. The shaded regions in (**b**) and (**d**) represent the s.d. of the T* obtained from the fit^26^. The error bars in (**e**) represent the s.d. obtained from the fit.

First we present the photodestruction of the pseudogap state measured by conventional two-pulse experiments. The photoinduced reflectivity (Δ*R*/*R*) below T* (determined in accordance with tunnelling experiments)^26^ ubiquitously shows two relaxation components (Fig. 2a). One is the PG response (which appears as a photoinduced decrease in reflectance, and whose amplitude we define as *A*_PG_) and the other arises from hot electrons energy relaxation (and appears as an increase in reflectance)^26^,^32^. The two components have different symmetry: the fast electron energy relaxation component has *A*_1*g*_ symmetry, while the pseudogap response has *A*_1*g*_+*B*_2*g*_ symmetry and are distinguished by Raman polarization selection rules^15^. The energy relaxation component does not change with temperature^26^, whereas *A*_PG_ gradually appears on cooling (Fig. 2a). In Fig. 2c we show the normalized fluence dependence of (Δ*R*/*R*). Initially, at low fluence *F* both components increase linearly, so the initial few traces overlap when normalized. However, soon the negative PG component begins to saturate while the energy relaxation component remains linear with *F* up to the highest measured fluence of 500 μJ cm^−2^.

In Fig. 2f,g we plot the fluence dependence of the amplitude *A*_PG_ of the pseudogap response for different temperatures and dopings, respectively, normalized to the saturation value (here and further we plot the absolute value of the peak amplitude of the pseudogap component). We assume that linearity of the response and correspondingly density of photoexcited quasiparticles are preserved until all the carriers forming the state are excited, that is, the pseudogap is destroyed. Inhomogeneous excitation arising from the finite light penetration depth (see supplement to ref. 33 for details) rounds the kink in the fluence dependence. We define the threshold value of the fluence required to destroy the PG state as *F*_TH_. Fits to the data are shown by solid lines in Fig. 2f,g.

As shown in Fig. 2f, *F*_TH_ is not very temperature dependent. The value of Δ*R*/*R* at saturation falls with increasing temperature (shown in black squares in Fig. 2d) and follows the *T*-dependence of *A*_PG_ in the weak excitation regime (open circles in Fig. 2d). In Fig. 2g we plot the fluence dependence of *A*_PG_ for the three different doping levels. The fluence dependence is very similar for all doping levels, but differs in the threshold value, increasing monotonically with T*, and accordingly decreasing with doping (Fig. 2e). Estimated heating corresponding to the largest value of *F*_TH_=58 μJ cm^−2^ for underdoped sample is ∼6 K; hence, the effects we observe are clearly of non-thermal origin.

### Recovery of the pseudogap state

**Figure 3:**
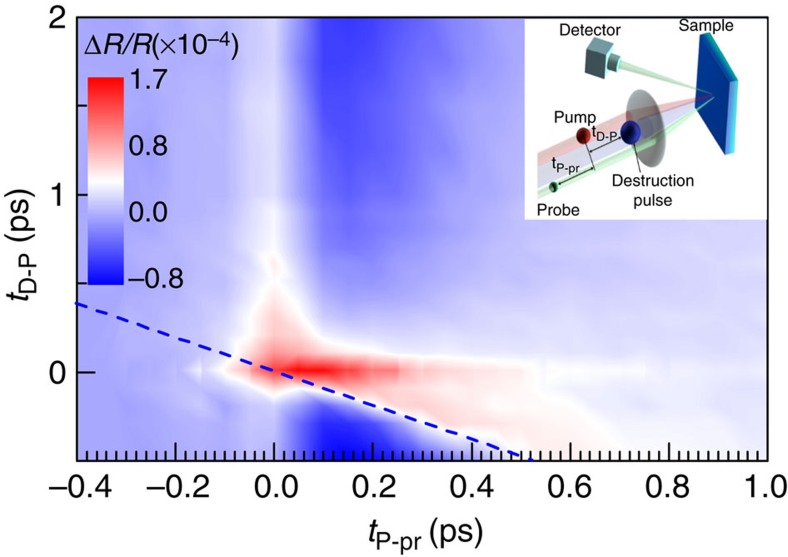
Typical result of the three pulse experiment. Underdoped sample at 120 K, the D pulse fluence is 204 μJ cm^−2^. The time of the D pulse arrival is shown by the dashed line. The D pulse suppresses the negative pseudogap component, whereas the hot electron energy relaxation response remains intact. Note that at later *t*_*D*−*P*_ the positive component is masked by the stronger negative PG component with a slower rise time. The inset shows a schematic picture of the three-pulse pump-probe experiment with the sequence of pulses and notation of delays. Colours of the pulses do not correspond to their photon energy.

To investigate the recovery of the pseudogap state, we measure the time-evolution of *A*_PG_ through the transition with the three-pulse technique. In this experiment we use an additional strong ‘destruction' (D) pulse (*F*_D_=14–204 μJ cm^−2^) to destroy the state and probe the evolution of quasiparticle response by performing the pump-probe (P-pr) experiment at variable delays between the D and P pulses. Homodyne detection is used to detect only changes in reflectivity caused by the pump pulse. Typical results of these experiments are shown in Fig. 3 for the UD sample.

**Figure 4:**
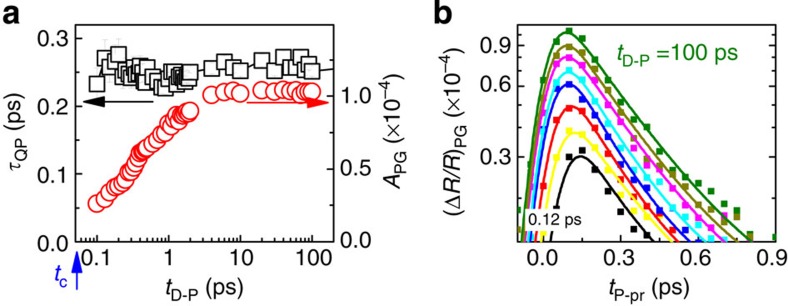
Evolution of amplitude and QP relaxation time. (**a**) Quasiparticle relaxation time (black squares, left axis) and the amplitude (red open circles, right axis) of the pseudogap component (data shown in **b**) as a function of *t*_D−P_. Quasiparticle relaxation time remains constant for all values of *t*_D−P_. The error bars represent s.d. obtained from the fit. (**b**) Pseudogap component for different values of *t*_D−P_ (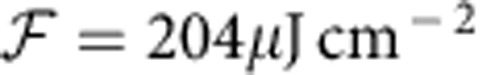, *T*=120 K).

First, we note that the hot electron energy relaxation response remains unaffected by the D pulse (also at room temperature, that is, above T*), and we can thus subtract it in all further data analysis. The pump-induced pseudogap response is suppressed at the moment when the D pulse arrives (shown by the dashed line). At later delays *t*_D−P_ we observe reappearance of *A*_PG_. In Fig. 4b we plot *A*_PG_ after the subtraction of the positive component, measured at the room temperature. The relaxation times obtained from the exponential function fit to the data are shown in Fig. 4a. Within experimental error, the PG relaxation time is constant throughout the PG recovery, at *τ*_PG_=0.26±0.05 ps. Remarkably, and in contrast to established behaviour in CDW^22^ and superconducting^23^ systems investigated so far, no critical behaviour of the QP response is observed anywhere near zero delay between D, P and p. (Even though we cannot determine the exact point of the transition in time, we can assume *t*_c_ to be approximately determined by the time that the electron energy relaxation process is over, near ∼50 fs (ref. 34).)

**Figure 5:**
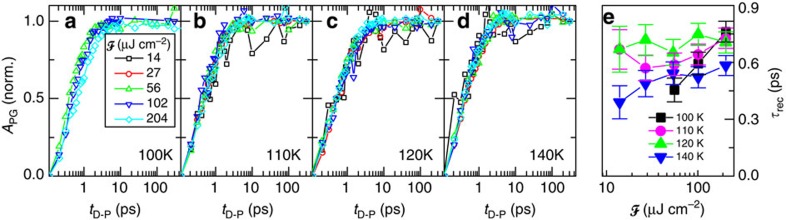
Recovery of the PG state after destruction by a laser pulse as a function of fluence at different temperatures. (**a**–**d**) The normalized amplitude of the pseudogap component as a function of *t*_D−P_ for different fluences at a number of temperatures is plotted. (**e**) The recovery time *τ*_rec_ as a function of fluence 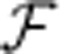 at different temperatures. The error bars represent s.d. obtained from fit with exponential function.

To compare the dynamics of the pseudogap recovery at different destruction fluences we plot in Fig. 5 the normalized *A*_PG_ as a function of *t*_D−P_, at different temperatures. Within the accuracy of the measurement the PG recovery time *τ*_rec_ is virtually independent of *F*.

Remarkably, for all temperatures and fluences, the PG recovery shown in Fig. 5 can be fit with an exponential function, which is not the case in the recovery of SC and CDW orders^22^,^23^, where the dynamical recovery behaviour associated with the formation of a collective state is more complicated. However, such a simple exponential recovery is consistent with uncorrelated dynamics of independent particles.

## Discussion

The distinct absence of critical behaviour as *t*→*t*_c_ in the PG state gives us new insight into the mechanisms for its formation. One possibility is that the normal to pseudogap transition is of first order. However, in this case we expect to observe an obvious step-like change of relaxation time at T* (ref. 35). Such effect has not been observed (see Fig. 2b), so a first order transition can be ruled out. Rather, the absence of a divergence in the QP excitation dynamics is a signature of finite size of the system either limited externally or just indicating a local nature of the states involved. The <50 fs resolution-limited uncertainty of the measured value of *τ*_QP_ allows us to put an upper limit on the correlation size (See Methods) of the pseudogap state to 
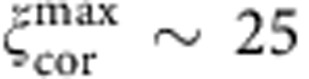
 Å, which is very close to the correlation length observed in diffraction experiments^12^.

A number of experiments suggest that the pseudogap is associated with bound (or localized) states^16^,^36^,^37^,^38^,^39^,^40^. A simple but plausible picture^24^ is that photoexcitation leads to the excitation of carriers from these states into itinerant states. Delocalization occurs most probably in the process of avalanche carrier excitation rather than during initial photon absorption. This is clearly seen from the slight delay of the negative pseudogap response with respect to the electron energy relaxation (see Fig. 3). Thereafter, binding takes place on a timescale given by *τ*_rec_ that is nearly independent on fluence and temperature, again indicating non-collective behaviour. This picture is supported by the fact that the pseudogap is filled rather than destroyed (that is, closed) after photoexcitation^41^, that is, a number of delocalized ‘in-gap states appear' without strongly altering the binding energy. This is tantamount to saying that the states do not act cooperatively, and there is no change of the energy scale, as the system evolves through *t*_c_ in time.

For a description of the fluence dependence of *A*_PG,_ the model which was widely used for the description of T-dependence of the pseudogap in low excitation regime is not appropriate^24^. In this model electrons and hot bosons with energy *ħ*Ω form a quasi-equilibrium with no change in the density of states. This can be approximately described in the framework of the two-level system (if *ħ*Ω≃Δ); however, in this case the density of photoexcited quasiparticles is expected to have a dependence on fluence of the form 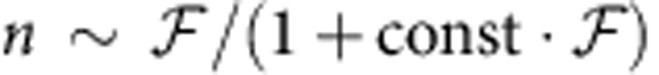. After inhomogeneity of excitation is taken into account, this becomes 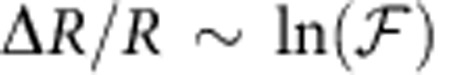 which predicts a much weaker dependence on 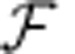 than observed experimentally (see Fig. 2f). In reality we clearly observe that initially the change in reflectivity and consequently *n* are linear with fluence.

In systems where photoexcitation of the gap-forming carriers is indirect, only part of the energy is used for the excitation of QPs and the destruction of the gapped state^42^. The total photodestruction energy is the sum of a lost energy *U*_lost,_ which arises from excitation of sub-gap phonons that is a monotonic function of the gap, and a much smaller binding energy for excitation across the gap *U*_bind_=Δ·*n*^42^. For a temperature-independent pseudogap^4^ only *U*_bind_ would depend on temperature via the temperature dependence of *n*(*T*), while the lost energy *U*_lost_ is independent of temperature thus explaining the apparent temperature independence of the photodestruction energy. By increasing the doping level the gap linearly decreases leading to the decrease of both *U*_lost_ and *U*_bind_ contributing to the photodestruction energy as shown in Fig. 2e).

Regarding the mechanism for localization, CDW formation is conventionally attributed to a Fermi surface (FS) instability arising from wavevector nesting^14^. However, recent ARPES studies in Bi_2_Sr_2−*x*_La_*x*_CuO_6+*δ*_ have revealed that the wavevector of the observed modulation does not correspond to the nesting between parallel sheets of FS at the antinodes but rather to the vector connecting ‘endpoints' of ‘Fermi arcs', so apparently no ‘true' nesting takes place^12^. In contrast, very different physics is responsible for ‘stripe' textures (which may occur on a similar scale), which are usually considered to be a result of the competing interactions, such as microscopic strain caused by localized holes and the Coulomb repulsion between them^10^,^11^,^43^,^44^,^45^, or the hole kinetic energy competing with the Coulomb interaction within Hubbard or *t*−*J* models^17^,^46^,^47^,^48^. We speculate that, within such stripe models, a weak FS instability may act to additionally stabilize hole pairs, enhancing spatial charge modulations through Friedel oscillations. The degree of nesting of the states at the Fermi surface^49^ would add an additional material-specific component that has a direct effect on *T*_c_.

The emerging physical picture is one in which photoexcited—or doped—carriers, subject to mutual Coulomb interaction and lattice strain, localize into a textured broken symmetry state without long-range order^10^,^11^,^43^,^44^,^45^. Once the density of localized particles is sufficient, achieved either through doping or cooling, superconductivity emerges through phase coherence percolation and Josephson tunnelling^38^,^45^. The *k*-space signature observable in ARPES comes from the polaron-like symmetry-broken strings in real space, of lengths up to 
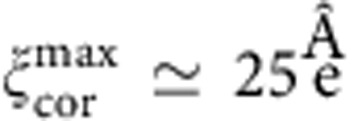
 along the Cu-O bond directions, which lead to an antinodal gap in reciprocal space^50^. (Fourfold symmetry is retained in ARPES because of spatial averaging). The extent of the PG in *k*-space is given by 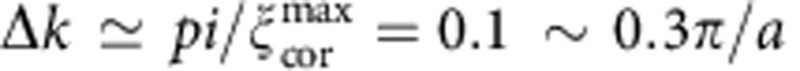 in agreement with experiments^1^. The presented picture is consistent with the electronic Raman scattering^15^,^51^ as well as with recent time-resolved ARPES^41^,^52^,^53^ where in the fluence dependence and the quasiparticle relaxation time in the antinodal pseudogap region are distinct from that of nodal region. A localization origin for the PG is also in agreement with tunnelling, specific heat and NMR data^1^.

To conclude, the data presented here, particularly the behaviour of the PG relaxation time through the coherently excited dynamical transition in Fig. 4 and exponential PG recovery dynamics in Fig. 5 imply a pseudogap state characterized by a short-range correlated localized carriers, pairs or very small clusters, locally breaking rotational symmetry^15^, rather than proper charge density wave segments^14^. The observations in Bi2212 are thus more consistent with a polaronic picture than a dynamically fluctuating charge density wave with long-range order. Our experiments also clearly show that the character of the PG state does not change with doping. Only the energy associated for its destruction diminishes, reflecting the change of localization energy (and T*) with increasing screening.

## Methods

### The three-pulse technique

The pulse train of 50 fs 800 nm laser pulses from a Ti:sapphire regenerative amplifier with a 250-kHz repetition rate was used to perform pump-probe (P-pr) reflectivity measurements. For the three-pulse experiment each laser pulse was split in three: the strongest destruction (D) pulse was used to destroy the state while the evolution of the state was monitored by measuring the pump-probe response at different delays between the D and P pulse. In three-pulse measurements pump fluence is always kept below 4 μJ cm^−2^, and probe fluence was ∼1 μJ cm^−2^.

### Samples

Three samples with different doping levels were investigated in this work: under- (*T*_c_=81 K, T*=180 K), near optimally- (*T*_c_=85 K, T*=140 K) and over- (*T*_c_=80 K, T*=120 K) doped Bi2212 with hole concentrations *P*=0.14, 0.19 and 0.21, respectively. Samples were grown by the travelling solvent floating zone method. Their critical temperatures were obtained from susceptibility measurements, doping levels and pseudogap temperatures were estimated from previous studies^54^.

### Estimation of maximum correlation length

The fermi velocity *v*_*F*_∼150 nm ps^−1^^55^ is an absolute maximum^56^ speed of propagation of exchange bosons, giving a correlation length 
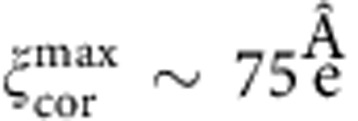
. A more precise estimate is based on a formula that links correlation length and relaxation time in the critical regime via corresponding diffusion constant 
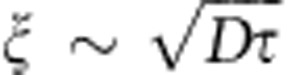
 (ref. 57), so that divergence of corresponding quantities is given by uncertainty of the experimental estimate 
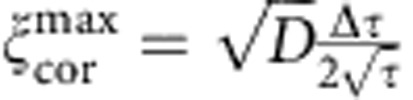
. *D* in turn can be estimated from measurable experimental parameters *v*_F_ (ref. 55) and normal state conductivity *σ* (ref. 58): 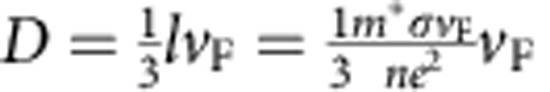. This estimate gives 
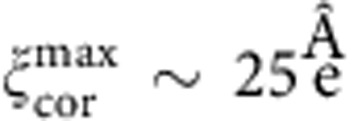
.

## Author contributions

T.K., Y.T. and M.O. has grown the samples and done magnetic characterization, I.M. did optical measurements and performed data analysis. I.M., T.M. and D.M. interpreted the data. I.M and D.M. wrote the manuscript.

## Additional information

**How to cite this article:** Madan, I. *et al*. Evidence for carrier localization in the pseudogap state of cuprate superconductors from coherent quench experiments. *Nat. Commun.* 6:6958 doi: 10.1038/ncomms7958 (2015).

## Figures and Tables

**Figure 1:**
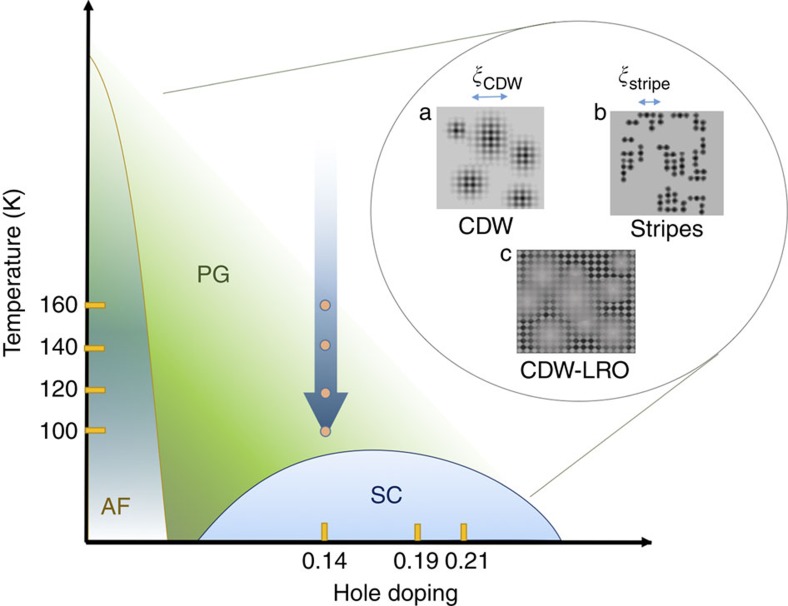
Phase diagram. A simplified phase diagram highlighting the different candidates most often discussed for the PG state: (**a**) a CDW-like state with spatial phase and amplitude fluctuations, (**b**) a stripe-like order arising from competing interactions, or (**c**) a long-range ordered (LRO) macroscopically coherent state with spatial amplitude fluctuations, or non-ordered areas^7^,^8^. The arrow shows the quench path. The orange dots schematically represent the base temperatures of the quench. Antiferromagnetic (AF) and superconducting (SC) phases are also shown. The doping level corresponding to the three samples investigated here is also shown.
